# COVID-19 in German Competitive Sports: Protocol for a Prospective Multicenter Cohort Study (CoSmo-S)

**DOI:** 10.3389/ijph.2022.1604414

**Published:** 2022-02-07

**Authors:** Andreas Michael Niess, Manuel Widmann, Roman Gaidai, Christian Gölz, Isabel Schubert, Katty Castillo, Jan Philipp Sachs, Daniel Bizjak, Shirin Vollrath, Fritz Wimbauer, Azin Vogel, Karsten Keller, Christof Burgstahler, Anne Quermann, Arno Kerling, Gerald Schneider, Jonas Zacher, Katharina Diebold, Maximilian Grummt, Claudia Beckendorf, Johannes Buitenhuis, Florian Egger, Andreas Venhorst, Oliver Morath, Friedrich Barsch, Klaus-Peter Mellwig, Julian Oesterschlink, Jan Wüstenfeld, Hans-Georg Predel, Peter Deibert, Birgit Friedmann-Bette, Frank Mayer, Anja Hirschmüller, Martin Halle, Jürgen Michael Steinacker, Bernd Wolfarth, Tim Meyer, Erwin Böttinger, Marion Flechtner-Mors, Wilhelm Bloch, Bernhard Haller, Kai Roecker, Claus Reinsberger

**Affiliations:** ^1^ Department of Sports Medicine, University Hospital of Tübingen, Tübingen, Germany; ^2^ Department of Sports and Health, Institute of Sports Medicine, Paderborn University, Paderborn, Germany; ^3^ Institute of Medical Informatics, Statistics and Epidemiology, School of Medicine, Technical University of Munich, Munich, Germany; ^4^ Hasso Plattner Institute, Digital Health Center, University of Potsdam, Potsdam, Germany; ^5^ Division of Sports and Rehabilitation Medicine, Department of Internal Medicine, University Hospital of Ulm, Ulm, Germany; ^6^ Department of Prevention and Sports Medicine, Center for Sports Cardiology/EAPC, School of Medicine, University Hospital “Klinikum Rechts der Isar”, Technical University of Munich, Munich, Germany; ^7^ Department of Cardiology, Cardiology I, University Medical Center Mainz (Johannes Gutenberg-University Mainz), Mainz, Germany; ^8^ Medical Clinic VII, Department of Sports Medicine, University Hospital Heidelberg, Heidelberg, Germany; ^9^ Institute of Sports Medicine, Hannover Medical School, Hannover, Germany; ^10^ Department I—Preventative and Rehabilitative Sports and Performance Medicine, Institute of Cardiology and Sports Medicine, German Sports University Cologne, Cologne, Germany; ^11^ Department of Sports Medicine, Charité—Universitätsmedizin Berlin and Humboldt-Universität zu Berlin, Berlin, Germany; ^12^ Center of Sports Medicine, University Outpatient Clinic, Potsdam, Germany; ^13^ Institute for Sport and Preventive Medicine, Saarland University, Saarbrücken, Germany; ^14^ Institute for Exercise and Occupational Medicine, Department of Medicine, Faculty of Medicince, Medical Center University of Freiburg, University of Freiburg, Freiburg im Breisgau, Germany; ^15^ Clinic for General and Interventional Cardiology/Angiology, Herz- und Diabeteszentrum NRW, Ruhr-Universität Bochum, Bad Oeynhausen, Germany; ^16^ Institute for Applied Training Science, Leipzig University, Leipzig, Germany; ^17^ Department of Orthopedics and Traumatology, University Medical Center Freiburg, Freiburg, Germany; ^18^ Department of Molecular and Cellular Sports Medicine, German Sport University, Cologne, Germany; ^19^ Institute for Applied Health Promotion and Exercise Medicine (IfAG), Furtwangen University, Furtwangen, Germany

**Keywords:** COVID-19, exercise, SARS-CoV-2, athletes, return to sport, myocarditis

## Abstract

**Objective:** It is unclear whether and to what extent COVID-19 infection poses health risks and a chronic impairment of performance in athletes. Identification of individual health risk is an important decision-making basis for managing the pandemic risk of infection with SARS-CoV-2 in sports and return to play (RTP).

**Methods:** This study aims 1) to analyze the longitudinal rate of seroprevalence of SARS-CoV-2 in German athletes, 2) to assess health-related consequences in athletes infected with SARS-CoV-2, and 3) to reveal effects of the COVID-19 pandemic in general and of a cleared SARS-CoV-2 infection on exercise performance. CoSmo-S is a prospective observational multicenter study establishing two cohorts: 1) athletes diagnosed positive for COVID-19 (cohort 1) and 2) federal squad athletes who perform their annual sports medical preparticipation screening (cohort 2). Comprehensive diagnostics including physical examination, laboratory blood analyses and blood biobanking, resting and exercise electrocardiogram (ECG), echocardiography, spirometry and exercise testing added by questionnaires are conducted at baseline and follow-up.

**Results and Conclusion:** We expect that the results obtained, will allow us to formulate recommendations regarding RTP on a more evidence-based level.

## Introduction

The current coronavirus pandemic is already one of the biggest global crises and poses an extreme challenge not only for the healthcare systems but is also a real test for social solidarity. Although the infection with severe acute respiratory syndrome coronavirus 2 (SARS-CoV-2) leads in the majority of the cases to light, moderate or even no symptoms it can also have severe to fatal consequences. Current knowledge of this disease shows that not only the lungs but also the cardiovascular system, the skeletal muscle, the central as well as the peripheral nervous system, the blood and the immune system, the liver, the kidneys and further body functions might be affected [1–6].

According to current knowledge, athletes are not a risk group predisposed for a severe course of COVID-19 disease. However, this fact does not prevent athletes from infection with SARS-CoV-2 that could be associated with moderate or even stronger acute symptoms [7]. In this context, more severe courses have been described in, prior to infection, fit and healthy athletes. Myocardial involvement in terms of myocarditis, as described as a possible cardiovascular sequela in COVID-19 [2], requires special attention in athletes not only with respect to return into competitive sport (return to play, RTP). In a small case sample of 26 US college athletes, who were tested positive for SARS-CoV-2 and presented with none or only mild symptoms, four males met criteria for myocarditis in cardiac magnetic resonance imaging (cMRI) [8]. In addition, a study including 789 North American professional athletes, who were tested positive for SARS-CoV-2, reported abnormal findings in 30 cases during the cardiac screening, whereas five of these were diagnosed with inflammatory heart disease (myocarditis or pericarditis) by additionally performed cMRI [9]. In a group of 90 young adult competitive athletes, cardiac consequences of SARS-CoV-2 has been described in 3.3% of the cases with one athlete with myopericarditis and two cases with pericarditis [10]. In a larger cohort of collegiate athletes, the prevalence of clinical myocarditis as assessed on a symptom-based diagnostic algorithm was 0.3%. In the same cohort, additional MRI revealed that 2.3% of the athletes met criteria for clinical and subclinical myocarditis [11]. Additional data showed a slightly lower prevalence (1.5%) in young competitive athletes after COVID-19 as detected by primary cMRI [12]. However, the true prevalence as well as the clinical relevance of these findings are not completely clear at present [13].

In a meta-analysis, exercise-induced dyspnea has been described as one of the leading symptoms, persisting longer than 2 weeks after COVID-19 diagnosis [14]. Initial data in the actual pandemic reveal a relevant percentage of COVID-19 patients with persisting respiratory symptoms and an impairment of pulmonary function longer than 100 days after initial diagnosis [15]. With respect to athletic performance, it must be taken into account that even minor restrictive changes might limit maximum ventilation and/or disrupt respiratory economy. In addition, a disturbance of gas exchange as a result of a diffusion disturbance is likely to restrict exercise tolerance and performance. Furthermore, there is preliminary evidence of a compromised oxygen transport to the consuming tissues, which occurs at the erythrocyte level [16].

In addition to cardiac, pulmonary and other sequelae, COVID-19 has been shown to be at risk for persistent symptoms and delayed recovery, actually termed as “long-COVID-syndrome” [17]. A meta-analysis, which assessed more than 50 long-term effects extending the acute phase of COVID-19 showed that 80% of COVID-19 patients suffered from at least one symptom persisting more than 2 weeks after initial diagnosis [14]. Persisting symptoms with highest frequencies were fatigue (58%), headache (44%), attention disorder (27%), hair loss (25%) and dyspnoea (24%). It is assumed that at least 10% of the COVID-19 survivors develop a long-COVID-syndrome, which may underestimate the real prevalence [18]. To date it remains speculative, how many of the athletes are affected and to what extent “long-COVID-syndrome” is present in physical active subjects and in particular competitive athletes, exhibiting only mild or no symptoms during the acute phase of the infection.

The complex and at this time point incompletely understood clinical picture of COVID-19 disease leads to the question of how reintegration into sport can be achieved with an acceptable risk after overcoming the acute phase of the COVID-19 infection [19]. For this purpose, a number of recommendations have been published [2, 20–22], which summarize primarily expert consensus statements. Thus, for the development of more evidence-based RTP concepts there is a need for extensive research in this field [23]. Because of the current uncertainty of long-term course of COVID-19 disease, the same is true with respect to the necessity of long-term follow-ups in affected athletes [24].

From today’s perspective, it is unclear whether and to what extent a mild or even asymptomatic COVID-19 infection poses an acute health risk and a more chronic impairment of athletic performance in competitive athletes. The identification and envisaged quantification of such impairments are an important decision-making basis for the management of the pandemic risk of this infection in sports and for athletes’ reintegration and return into competitive sports. In this context, it is important to note that even a slight decrease in physical performance, not perceivable in daily living, may compromise competitive success in elite athletes. Nevertheless, to delineate subtle perturbations of COVID-19 disease on exercise performance and its underlying pathophysiology may also have importance for the non-athletic population. With respect to the prevalence of COVID-19 in competitive athletes, reliable epidemiological data is still lacking. Since asymptomatic or oligosymptomatic courses are presumably common in younger people [12], a high number of unreported cases of passed infections has to be expected, also and maybe particularly among competitive athletes. Nevertheless, it is unclear whether the prevalence of COVID-19 infection in competitive athletes differs from that of the general population in the same age-group. Moreover, it is unclear, whether there may be differences within the athlete population, depending on the type of sports and performance level, the grade of intensive physical contacts and the extent of sports-related travel activities. As described, the COVID-19 pandemic represents one of the greatest challenges also for competitive sports and, in particular, its sports medicine monitoring. In our opinion, this challenge can only be addressed in a multicentric approach. The objectives of our project are to investigate open questions about the type and frequency of potential health risks and remaining symptoms, to assess consequences of this disease on athletic performance and to determine the seroprevalence of COVID-19 diseases in the athletic population. In this context, close networking of the sports medicine centers in Germany is also useful against the background that although the infection rates exhibit intermediate drops and vaccination rates increase, the occurrence of virus mutations [25] and an unclear duration of immunity after vaccination clearly reflect that the pandemic is still in progress.

## Methods

### Central Research Questions

This study aims 1) to analyze the longitudinal rate of seroprevalence of SARS-CoV-2 in national squad athletes, 2) to assess acute and prolonged health-related consequences in athletes infected with SARS-CoV-2, and 3) to reveal effects of the COVID-19 pandemic in general and of a cleared SARS-CoV-2 infection on physical performance.

### Design and Setting of the Study

**FIGURE 1:**
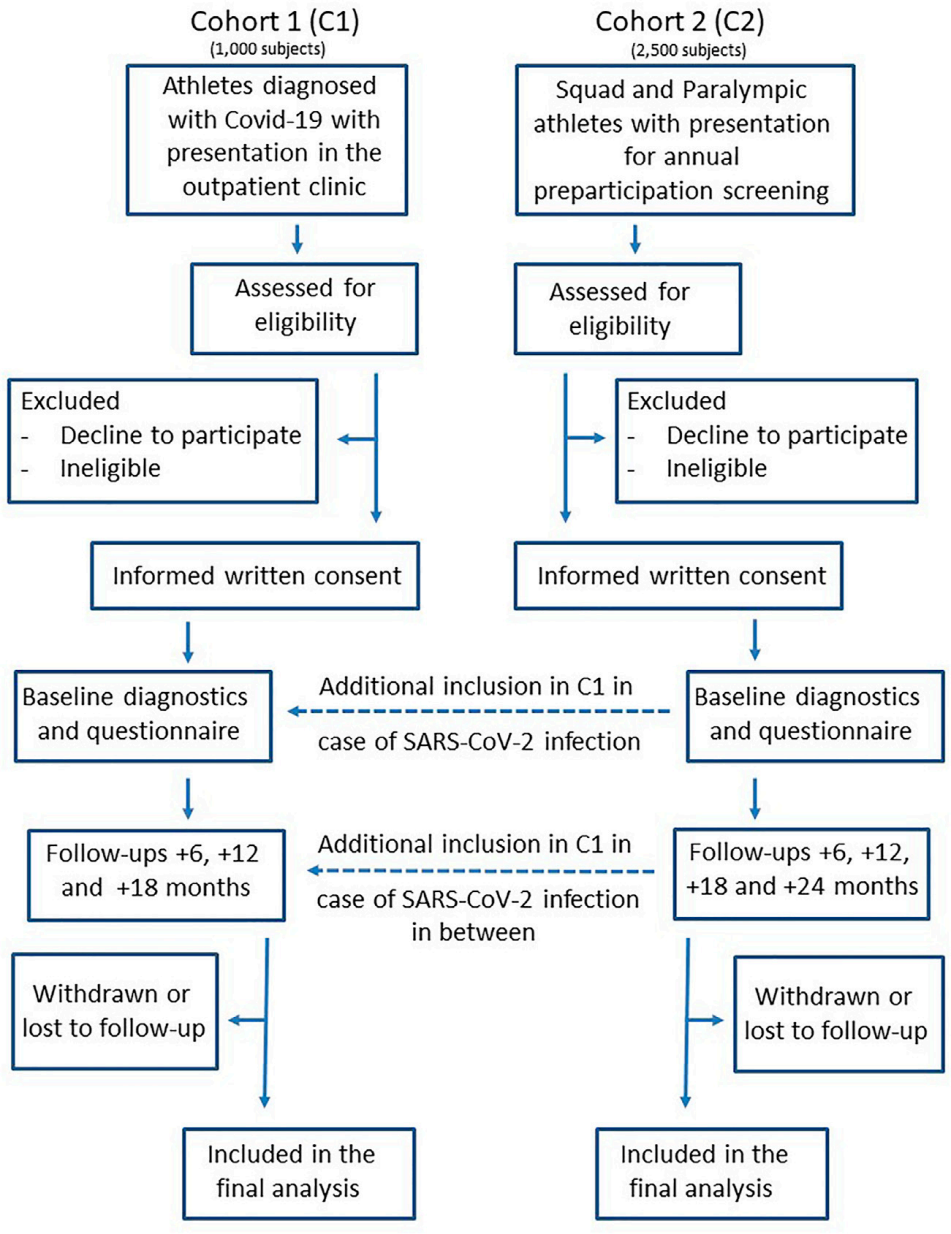
Prospective multicenter cohort study flow diagram of cohort 1 and cohort 2 (COVID-19 in German competitive sports: Protocol for a prospective multicenter cohort study; Germany, 2020).

CoSmo-S (COVID-19 in elite sports—A multi-center cohort study) is organized as a prospective observational multicenter hybrid cohort study by establishing and investigate two study cohorts: 1) Athletes diagnosed positive for Covid-19 disease (cohort 1, C1) and 2) federal squad athletes and Paralympic athletes* who routinely present themselves for their annual sports medical preparticipation screening (cohort 2, C2) (Figure 1).

Examinations are performed in 13 sports medical outpatient clinics in Germany, licensed from the German Olympic Association (DOSB) for preparticipation screenings in federal squad athletes and Paralympic athletes. Most of the outpatient clinics are located at universities. In both cohorts the athletes receive comprehensive diagnostics at baseline (*t*_0_), as well as follow-up (C1: +1 year, C2: +1 and +2 years), including medical history, physical examination, laboratory blood parameters, resting and exercise ECG, echocardiography, spirometry and exercise testing added by questionnaires for anamnesis and on COVID-19—specific aspects such as type and duration of symptoms (additionally +6 and +18 months), respectively. Biobanking is performed on plasma and serum samples as well as optionally on isolated peripheral blood mononuclear cells (PBMC) for further analyses (for details see Tables 1, 2).

**TABLE 1:** Schedule of diagnostic data collection in Cohort 1 and Cohort 2 in the prospective multicenter cohort study (COVID-19 in German competitive sports: Protocol for a prospective multicenter cohort study; Germany, 2020).

	Cohort 1				Cohort 2				
Time point	*t*_0_	*t*_1_	*t*_2_	*t*_3_	*t*_0_	*t*_1_	*t*_2_	*t*_3_	*t*_4_
Month	0	6	12	18	0	6	12	18	24
Assessments									
Screening and study inclusion	X				X				
Questionnaires									
COVID-19 specific questionnaire									
- Prior/intermediate SARS-CoV-2 testing	X	X	X	X	X	X	X	X	X
- Contact situations with SARS-CoV-2 positive subjects	X	X	X	X	X	X	X	X	X
- Prior/intermediate acute symptoms of infection	X	X	X	X	X	X	X	X	X
- Experience of unusual/chronic symptoms	X	X	X	X	X	X	X	X	X
- Necessity and duration of quarantine	X	X	X	X	X	X	X	X	X
- SARCS-CoV-2 vaccination state	X	X	X	X	X	X	X	X	X
- Actual exercise tolerance and performance	X	X	X	X	X	X	X	X	X
Anamnesis									
- Medical history	X		X		X		X		X
- Training report	X	X	X		X	X	X	X	X
- Family history	X		X		X		X		X
- Medication	X		X		X		X		X
- Dietary habits/dietary supplements	X		X		X		X		X
Medical examination									
Anthropometry									
- Height, weight, body mass index	X		X		X		X		X
- Percent body fat	X		X		X		X		X
Physical examination	X		X		X		X		X
Blood sampling and analyses									
Blood analyses									
- Blood cell and differential cells counts, C-reactive protein, liver enzymes, creatinine, ferritin, troponin^a^,	X		X		X		X		X
d-dimers^a^, urine protein			X		X		X		X
- SAR-CoV-2 antibody testing	X		X		X		X		X
- Biosampling (plasma and serum)	X		X		X		X		X
- Isolation of PBMC^b^ (optional)	X		X		X		X		X
Cardiorespiratory diagnostics									
Twelve-lead-resting electrocardiogram (ECG)^c^	X		X		X		X		X
Resting blood pressure	X		X		X		X		X
Resting spirometry									
- Static lung variables^d^	X		X		X		X		X
- Dynamic lung variables^e^	X		X		X		X		X
Echocardiography									
- Morphology, left and right heart dimensions^f^	X		X		X		X		X
- Left and right heart systolic and diastolic function^g^	X		X		X		X		X
- Strain analysis (optional)	X		X		X		X		X
- Doppler analysis^h^	X		X		X		X		X

a Only if individually indicated.

b PBMC, peripheral blood mononuclear cells.

c Interpretation of the ECG of athletes according to [26].

d Inspiratory vital capacity (IVC), Forced vital capacity (FVC).

e One-second vital capacity (FEV1), Tiffenau–index (FEV1/FVC), peak exspiratory flow (PEF), maximum exspiratory flow at 75% FVC (FEF75%) and 25–75% FVC (FEF25-75%).

f Left ventricular enddiastolic diameter (LV-EDD), left ventricular endsystolic diameter (LV-ESD), Right ventri-cular enddiastolic diameter (RV-EDD), mode), enddiasystolic volume (EDV), endsystolic volume (ESV) left and right atrial dimensions (planimetric and M-mode), heart volume (HV, absolute and relative). Basal right ventricular diameter (RVD1), systolic septal thickness (LV-STs), diasystolic septal thickness (LV-STd), systolic posterior wall thickness (LV-PWs), diastolic systolic posterior wall thickness (LV-PWs), LV mass (LVM), LV mass index (LVMi), signs and dimension of pericardial effusion.

g Fractional shortening (FS), ejection fraction (EF, by ESD and EDV, according Simpson), regional wall motion, tricuspid annular plane systolic excursion (TAPSE), E-wave, A-wave, E/A-ratio, E′-wave septal, E′- wave lateral and E/E′– wave ratio.

h Colour-, PW- and CW-doppler of heart valves, maximum systolic pulmonal artery pressure *via* maximum tricuspidal regurgitation velocity (maximum PAPsys).

**TABLE 2:** Schedule of ergometric data collection in Cohort 1 and Cohort 2 in the prospective multicenter cohort study (COVID-19 in German competitive sports: Protocol for a prospective multicenter cohort study; Germany, 2020).

	Cohort 1				Cohort 2				
Time point	*t*_0_	*t*_1_	*t*_2_	*t*_3_	*t*_0_	*t*_1_	*t*_2_	*t*_3_	*t*_4_
Month	0	6	12	18	0	6	12	18	24
Exercise testing									
Incremental exercise test^a^									
- Twelve-lead-electrocardiogram	X		X		X		X		X
- Blood pressure	X		X		X		X		X
- Capillary blood lactate^b^	X		X		X		X		X
- Rate of perceived exertion (BORG-scale)^c^	X		X		X		X		X
- Pulse oximetry	X		X		X		X		X
- Breath gas analysis/Spiroergometry (optional)^d^	X		X		X		X		X
Additional ramp test (if possible)^e^									
- Breath gas analysis/Spiroergometry^f^	X		X						
- Capillary blood lactate (pre/post exercise)	X		X						
- Capillary blood gases	X		X						

a Treadmill, bicycle, canoe or rowing ergometry with stepwise increase of exercise load until voluntary exhaustion. The incremental exercise test is performed in both cohorts.

b Calculation of exercise load at the lactate threshold (LT), individual anaerobic threshold (IAT) and at the end of exercise from the lactate-performance curve.

c Assessment of perceived exertion (RPE) pre-exercise, at the end of every stage during the test and post-exercise according the qualities “overall,” “muscular,” and “ventilatory.”

d Additional breath gas analysis is performed in cases where breath gas analysis was also performed in an existing previous test.

e An additional ramp test with breath gas analysis is performed only in C1 provided that the athletes tolerate a second test. Protocol: bicycle ergometry, starting at an initial load of .5 W per kilogram body mass with a continuous increment of .5 W per kilogram body mass until voluntary exhaustion.

f Measures: maximum oxygen consumption (VO2max), ventilatory threshold 1 (VT1), respiratory compensation point (RCP), maximum minute ventilation (VEmax), tidal volume, oxygen pulse, ventilatory equivalents (VE/VO2 and VE/VCO2), endtidal VCO2 (ET- VCO2).

In C1, diagnostic is expanded by breath gas analysis and—if clinically indicated—by further diagnostics such as cMRI, lung computed tomography (CT) or assessment of lung diffusion capacity. Moreover, pre-existent diagnostic findings related to the actual SARS-CoV-2 infection of the individuals in C1 are documented. In C2, data from previous exercise testing and resting spirometries, mostly available from the last preparticipation screening prior to the COVID-19 pandemic, are also included. To ensure comparability of data from exercise testing, it is recommended to use the identical exercise testing protocols at the post-COVID-19 test for each subject as performed in the pre-COVID-19 examination.

After 6 months (*t*_1_), initial diagnostics in C1 and C2 are followed by a web-based questionnaire addressing follow-up medical history and COVID-19 specific aspects. One year post-baseline, a follow-up examination (*t*_2_) including the diagnostic procedures from *t*_0_, is planned for each participant. In turn, web-based questionnaire is repeated at 18 months (*t*_3_) in C1 and C2 and another examination will take place at 24 months (*t*_4_) only in C2.

### Characteristics of the Participants

#### Inclusion Criteria

In C1 inclusion criteria comprise an age of at least 18 or older, proven infection with SARS-CoV-2 assessed by 1) positive swap (polymerase chain reaction, PCR) or 2) positive serum SARS-CoV-2 IgG plus 3) typical acute symptoms and 4) ambiguous sport activities with a minimum of three training sessions per week corresponding to a minimum energy expenditure of 20 MET hours per week. Inclusion criteria of C2 are an age of 14 years or older, being member of the German federal squad or Paralympic federal squad and presentation for annually routine preparticipation screening at one of the DOSB licensed study centers. In both cohorts, inclusion requires a written informed consent of all participants. If the participants in C2 are not aged 18 years or older, the written consent has to be additionally signed by the agreeing parents of the participant. If a participant of C2 develops COVID-19 during the course of the study and is 18 years or older, this athlete is also included in C1.

#### Exclusion Criteria

Exclusion criteria in C1 and C2 are acute COVID-19 infection with a positive SARS-CoV-2 PCR within the last 2 weeks, refusal of venous blood sampling, insufficient skills of German language, acute or chronic disease that does not allow inclusion as estimated by the study physician or withdraw of the agreement to participate in the study.

### Data Collection

A large part of the data is collected as part of routine diagnostics as recommended prior return to sports and/or to clarify persistent exercise intolerance after COVID-19 disease [20] in C1 and as defined for the preparticipation screening program of the DOSB for squad athletes [27] in C2. Study specific data collection and diagnostics include a COVID-19 specific questionnaire, SARS-CoV-2 antibody testing and biobanking of plasma and serum samples. Since the national squad athletes present themselves annually at the DOSB-licensed centers as part of the annual examinations, there are in the majority of athletes examination results from the pre-pandemic time available. These allow a more precise assessment of intra-individual comparison between pre- and post-COVID-19 examination results of the same person and to delineate possible effects of the COVID-19 pandemic on physical performance.

### Data Management

All data are primarily documented in the original patient charts of the athletes and then transferred to the electronic CRF (eCRF) of the data capture system REDCap (Research Electronic Data Capture) [28, 29] hosted at Paderborn University. REDCap data entry is implemented following the diagnostic structure outlined in Tables 1, 2 using repeatable instruments. Validation of data entry is implemented where applicable. Access to patient data and data entry is restricted to the respective study center by means of data access groups corresponding to the 13 study centers. Data transfer to and from REDCap is fully encrypted.

In the initial phase of the study, COVID-specific questionnaires are paper-based and detached in the course by a tablet—based solution, which automatically transfers the results to the REDCap data base. Tablet and web-based questionnaires are implemented utilizing REDCap’s survey function. For missing data, no assumptions are made. After enrollment into the study, the athletes were coded with an eight-digit alphanumerical code under which all entries will be made in the database and which can only be assigned at the respective study center. In order to reduce an investigator-specific bias on the interpretation of exercise test data, all raw data from exercise tests are pooled and then evaluated in a standardized manner according to identical calculation criteria.

### Quality Aspects and Monitoring

Upon initialization of the study, all study centers are briefed on the data entry by the IT-team of the study. During the initial process of the implementation, minor adjustments of the data base are done to further improve the process of data entry. An internal monitoring is implemented to ensure valid data transfer and documentation in the data capture system REDCap. Quality checks of the data are performed and allow the generation of queries. Moreover, monitoring includes randomly organized visits to single study centers to check for the validity of the data transfer to the data capture system. Missing data are labelled as N/A or N/D and, if appropriate, added by a comment.

### Statistical Analysis

For relevant categorical variables as e.g. documented symptoms experienced during the COVID-19 infection, persistent symptoms, presence/absence of antibodies or quarantine measures absolute and relative frequencies will be presented. To investigate associations of infection characteristics, patient characteristics or sports-related characteristics on prolonged recovery after COVID-19 infection and delayed return to sports odds ratio will be calculated and Chi-square tests will be performed trying to identify potential risk factors for COVID-19 infection, long absence from training and competition, and long-lasting or severe symptoms. For continuous outcomes, means and standard deviations or medians and interquartile ranges will be calculated. Multivariable regression models will be fitted to the data to consider multiple independent variables simultaneously and to account for potential confounders. All statistical tests will be performed two-sided on a significance level of *α* = 5%. For relevant measures 95% confidence intervals will be estimated.

In both cohorts, sample size was not formally calculated. In C1, recruitment numbers depend on the development of the pandemic, but we estimate a minimum of 1,000 athletes, who will be included into C1. In C2, reaching the proposed minimum number of 2,500 federal squad athletes and Paralympic athletes means, that at least 62% of all German squad athletes will be included in CoSmo-S. Actually, we have defined no upper limit for athlete´s recruitment in C1 and C2.

### Machine Learning-Based Outcome Predictions

Clinical predictive models rely on machine or deep learning (ML/DL) methods. Unlike their more traditional application on retrospective data (such as electronic health records and billing data), prospectively collected data such as in this project have comparatively low percentages of missing and erroneous values, increasing the predictive performance without data imputation. Clinically significant outcome variables such as the physical performance or other health-related consequences will be binarized and serve as target variables, while the other data will become feature variables. The actual models can either be time-insensitive (e.g., traditional ML models, such as XGBoost or Random Forest) or consider the longitudinality of the observations (e.g., deep learning architectures, such as long short-term memory (LSTM) networks). The input data will be split into training, validation, and test sets. All modeling results will undergo a feature importance analysis to assess the impact of individual features on the prediction performance.

### Ethics Approval and Consent to Participate

Ethics approval was obtained by the “Ethics Committee of the Medical Faculty, University of Tübingen” on the 28 July 2020 (reference number: 608/2020BO1). The work described is carried out in accordance with the Declaration of Helsinki for experiments involving humans. All participants are informed by the study physician about the study procedure, subsequent data storage, and confidentiality and anonymity regarding the data. Written informed consent is collected from all participants or their parent or legal guardian in the case of an age under 18 years that the study centres are allowed using the data for research analyses and publishing the data by using a study consent form. The study has been registered in the German Clinical Trials Register (DRKS00023717).

### Trial Status

Recruitment of athletes started at 1st October 2020 and will last until 30 June 2022. Actually (mid of July 2021) 429 athletes were successfully recruited in C1 and 735 in C2, respectively.

## Results and Discussion

As the COVID-19 pandemic still affects competitive sports all over the world, knowledge of its impact on health of the athletes is still sparse, which emphasizes the urgent need for the present cohort study. If we are successful in recruiting more than 1,000 subjects in C1 and 2,500 or more in C2, CoSmo-S will represent the largest study in competitive athletes on this field so far in Germany. Reaching this sample sizes will allow us to perform substantial subgroup analyses that might provide valuable information on potential sports associated factors for risk of COVID-19 infection or mid-to long-term consequences for competitive athletes. Moreover, in these well characterized two cohorts, additional in-depth research can be conducted to address more specific questions such as effects of COVID-19 on T cell function. This should help to reveal the extent and magnitude of potential detrimental effect of SARS-CoV-2 infection even in athletes and physical active people. Additional subgroup analyses may be performed with respect to the started vaccination program against SARS-CoV-2 in the athlete population.

One key strength of our cohort study is that pre-study examination results are available from the majority of the squad athletes in C2 and in a lower frequency also in C1. This allows not only a more accurate detection of the effects of the pandemic on the ergometric performance of the athletes but also a more subtle assessment of pulmonary function, e.g., by comparing flow-volume curves in the spirometry with individual findings from the pre-pandemic time.

As we measure objective physical fitness by standardized ergometry, we can link these fitness data with initial and probably ongoing symptoms in the post-COVID cohort (C1). In turn, we hope to delineate predictors (e.g., symptom characteristics) for the individual risk to develop a drop in physical function and/or prolonged fatigue, which may also serve as a model for the general population. Moreover, negative effects of a COVID-19 disease on physical fitness and exercise tolerance are at risk to have detrimental effects on public health at all. As the recruited athletes individually perform a broad range of different types, volumes and intensities of training, it might be possible to assess the impact of different physical activity patterns on Long-COVID and to delineate underlying factors contributing to fatigue and other sequelaes. It is planned to compare our data with those obtained in other cohorts recruiting subjects from the general population.

Nevertheless, some limitations of the study merit consideration: In C1, athletes present themselves voluntarily to our outpatient clinics for post COVID-19 diagnostics and RTP examination, baring the risk for self-selection bias. It cannot be excluded that those athletes mainly present themselves in our clinics who have noticed acute symptoms or persistent complaints. In this context, it seems to be helpful that we can additionally calculate the percentage of symptom-free, but SARS-CoV-2 positive tested squad athletes also from C2. We hope that the results obtained in this cohort study will allow us formulate reliable recommendations regarding RTP on an evidence-based level.

## Data Availability

The datasets generated and/or analyzed during the current study are not publicly available because some of the data are from German top athletes and general public availability may lower the recruitment rates in our study. However, the datasets may be available from the corresponding author on reasonable request.
